# A Pectin-Rich, Baobab Fruit Pulp Powder Exerts Prebiotic Potential on the Human Gut Microbiome In Vitro

**DOI:** 10.3390/microorganisms9091981

**Published:** 2021-09-17

**Authors:** Martin Foltz, Alicia Christin Zahradnik, Pieter Van den Abbeele, Jonas Ghyselinck, Massimo Marzorati

**Affiliations:** 1 Döhler GmbH, 64295 Darmstadt, Germany; aliciachristin.Zahradnik@doehler.com; 2 Cryptobiotix SA, 9052 Ghent, Belgium; pieter.vandenabbeele@cryptobiotix.eu; 3 ProDigest BV, 9052 Ghent, Belgium; jonas.ghyselinck@prodigest.eu (J.G.); massimo.marzorati@prodigest.eu (M.M.); 4 Center of Microbial Ecology and Technology (CMET), Ghent University, 9000 Ghent, Belgium

**Keywords:** baobab fruit pulp powder, prebiotic, pectin, interindividual variation, in vitro, gut microbiota, dialysis

## Abstract

Increasing insight into the impact of the gut microbiota on human health has sustained the development of novel prebiotic ingredients. This exploratory study evaluated the prebiotic potential of baobab fruit pulp powder, which consists of pectic polysaccharides with unique composition as compared to other dietary sources, given that it is rich in low methoxylated homogalacturonan (HG). After applying dialysis procedures to remove simple sugars from the product (simulating their absorption along the upper gastrointestinal tract), 48 h fecal batch incubations were performed. Baobab fruit pulp powder boosted colonic acidification across three simulated human adult donors due to the significant stimulation of health-related metabolites acetate (+18.4 mM at 48 h), propionate (+5.5 mM at 48 h), and to a lesser extent butyrate (0.9 mM at 48 h). Further, there was a trend of increased lactate levels (+2.7 mM at 6h) and reduced branched chain fatty acid (bCFA) levels (−0.4 mM at 48 h). While *Bacteroidetes* levels increased for all donors, donor-dependent increases in *Bifidobacteria*, *Lactobacilli*, and *Firmicutes* were observed, stressing the potential interindividual differences in microbial composition modulation upon Baobab fruit pulp powder treatment. Overall, Baobab fruit pulp powder fermentation displayed features of selective utilization by host microorganisms and, thus, has promising prebiotic potential (also in comparison with the ‘gold standard’ prebiotic inulin). Further research will be required to better characterize this prebiotic potential, accounting for the interindividual differences, while aiming to unravel the potential resulting health benefits.

## 1. Introduction

The human gut harbors an enormous number of bacteria which strongly affect human health [1,2]. The vast majority of these bacteria reside in the colon with an abundance of approximately 10^11^ bacterial cells per mL of content [3]. Colonic bacteria ferment nutrients and fibers that are undigestible to the host, thereby producing a multitude of metabolites. Among these metabolites, the health-promoting short-chain fatty acids (SCFAs) acetate, propionate, and butyrate have received large interest in the past few decades [4,5]. The extent and the nature of metabolites produced strongly depend on the bacterial community composition, which is shaped by myriad parameters, with the diet being of utmost importance [6,7,8]. Currently, it is well understood that each human individual harbors a unique microbiome which interacts distinctly with the diet [9,10]. While there is a need for better controlling and registering the factors that drive the interindividual variation in gut microbiome composition [11], it is also crucial to include multiple human subjects when screening new ingredients to account for such interindividual differences. 

Increased understanding of the gut microbiota has triggered research toward improving human health through stimulation of beneficial gut bacteria. In this aspect, prebiotic substrates have gained a lot of attention [12,13]. While the definition of a prebiotic has evolved over the past decades, a consensus was recently reached defining prebiotics as substrates that are selectively utilized by host microorganisms conferring a health benefit [14]. While prebiotics can, thus, be administered to any host microbial ecosystem (e.g., vagina, skin), many prebiotic strategies have focused on dietary applications. In this respect, substrates such as inulin, fructooligosaccharides, and galactooligosaccharides are considered ‘gold standard’ given their well-documented effects on the gut microbiome. A potential issue of aforementioned prebiotics is their relatively rapid fermentation in the proximal colon, which could result in limited tolerance (e.g., bloating and abdominal pain) at high doses [15,16]. Such findings have stimulated the development of novel prebiotics including pectin-based poly- and oligosaccharides. Pectins are a group of complex heteropolysaccharides found in the cell walls of all plants and mainly consist of galacturonans (homogalacturonan (HG), substituted galacturonans, and rhamnogalacturonan-II) and rhamnogalacturonan-I (RG-I) [17,18]. Different combinations of these components, as well as variations within each component, allow for a wide range of pectic polysaccharides to be formed. The varying composition of monosaccharides and their length puts pectin-based fibers as potential prebiotics, as they need to be fermented with specific enzymatic machinery [19].

The baobab fruit is derived from the baobab tree (*Adansonia digitate* L.) indigenous to Africa, particularly Sudan, Ghana, Malawi, Burkina Faso, and Uganda, and it is a potential source of pectins. Baobab fruit is subdivided into pericarp, resistant outer shell, endocarp, and the inner ripe fruit. The ripe pulp is floury, dry, and powdery, including red fibrous structures and seeds. Although the nutritional values of baobab pulp vary among different regions, its fiber content is around 70–80% of its dry mass [20]. Despite abundant commercial claims on the health effects mediated by baobab, scientific data on its composition, potential mechanism of action, and effects are scarce. So far, only two preliminary studies have been reported investigating the effects of baobab in humans. In a single-blinded crossover study, the influence of 15 g baobab extract formulated into a smoothie was investigated in 20 subjects. Baobab exerted a reduced feeling of hunger, which was likely caused by the increased fiber content in the baobab treatment (11 g) as compared to the control (2 g) [21]. Furthermore, baobab fruit powder tested at two doses (18.5 g and 37 g) after white bread consumption significantly reduced postprandial blood glucose response when compared to the control treatment [22]. Lastly, in vitro studies on the prebiotic potential of baobab are also lacking.

Although in vivo studies are fundamental to demonstrate a health effect on the host, an important hurdle to understand the prebiotic modulation of the human gut microbiome in vivo is the limited access to the site of activity. The use of fecal samples in in vitro fermentation models might help in uncovering the enigmas of gut–bacterial interactions [23] and in evaluating and screening of novel potential prebiotics. While dynamic gut models have been applied before, the high reproducibility and especially higher throughput of short-term incubation models have been proposed to be critical for further broadening the understanding of the gut microbiome [24]. With respect to gaining an understanding of potential prebiotic effects of novel substrates, such models allow the inclusion of multiple test products, while simultaneously addressing interindividual differences among human subjects [25]. 

This study aimed to investigate the prebiotic potential of a novel ingredient rich in pectin-based polysaccharides (baobab fruit pulp powder). This ingredient is unique compared to other pectic polysaccharides given its high content of low methoxylated HG. A 48 h in vitro incubation strategy with the human fecal microbiota of three different human donors (to account for interindividual differences) was applied to investigate the potential modulation of microbial fermentation products (acidification, gas production, SCFAs, and branched chain fatty acids (bCFAs)) and levels of five specific taxonomic groups (*Bifidobacteria*, *Lactobacilli*, *Firmicutes, Bacteroidetes*, and *Akkermansia muciniphila*). To our knowledge, this is the first study demonstrating the potential of baobab fruit pulp powder to modulate the human gut microbiota.

## 2. Materials and Methods

### 2.1. Products

Baobab fruit pulp powder (BP) tested in the current study was provided by Döhler GmbH (Darmstadt, Germany) (Table S1). BP contains a soluble fiber fraction predominantly consisting of pectic polysaccharides (42.5% of dry mass). Pectic polysaccharides had a low degree of methylation (11%). Acetyl esterificiation of pectic oligosaccharides was found in trace amounts only. In other words, it mainly consisted of low methoxylated HG. Furthermore, BP contained an insoluble fiber fraction (13% of dry mass) consisting of hemicellulose, cellulose, and cell-wall material bound to pectin, starch (2.7% of dry mass), and proteins (2.8% of dry mass). Lastly, BP also comprised a substantial amount of glucose, fructose, and sucrose (30% of dry mass). Unless otherwise stated, all other chemicals were obtained from Carl Roth (Karlsruhe, Germany).

### 2.2. Dialysis of Test Product

**Figure 1:**
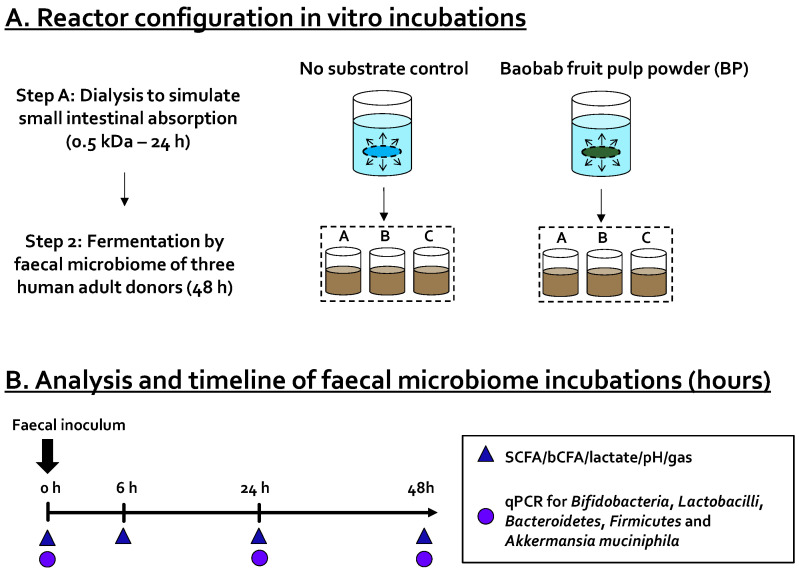
Schematic representation of the experimental design in this study to investigate the prebiotic potential of baobab fruit pulp powder (BP). (**A**) First, upper gastrointestinal absorption was simulated through dialysis of the baobab fruit pulp powder. Second, 48 h fecal batch incubations were performed to assess the prebiotic potential of the dialyzed baobab fruit pulp powder on fermentation products (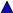) and levels of specific taxonomic groups via qPCR (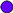) compared to ‘no substrate control’ incubations for three healthy adult donors. (**B**) Sampling scheme to evaluate the effect of the dialyzed baobab fruit pulp powder. SCFA = short-chain fatty acid, bCFA = branched-chain fatty acid, qPCR = quantitative polymerase chain reaction.

As glucose, fructose, and sucrose (present in BP) are absorbed in the small intestine in vivo, upper gastrointestinal absorption was simulated via a dialysis procedure. This allowed testing the relevant fraction of the test product that would reach the GI tract (Figure 1). Dialysis was performed as previously by Van den Abbeele et al. [26] with minor modifications. Briefly, 100 mL of a baobab fruit pulp powder suspension (64.4 g/L) was prepared in dH_2_O and introduced into a cylindrical dialysis membrane with a molecular weight cutoff of 0.5 kDa (Spectrum Europe BV, Paris, France). After sealing, the membrane was submerged in 600 mL of dialysis fluid (3.75 g/L NaHCO_3_ in dH_2_O; pH 7) for 24 h at 4 °C to prevent the growth of bacteria. During the dialysis procedure, sugars moved from the intestinal content to the dialysis suspension. On the other hand, due to osmotic pressure, water also moved from the dialysis solution toward the compartment simulating the intestinal content. This additional dilution of the test product was calculated by measuring both the initial (~100 g) and the final weight of the intestinal content. This dilution was then accounted for when adding the dialyzed product to the colonic incubations so that a fixed amount of test product was dosed, which was equivalent to 4 g of non-dialyzed test product/L colonic medium. In other words, when high quantities of water entered the intestinal content compartment, lower amounts of water were dosed at the start of the colonic incubation.

### 2.3. Short-Term Colonic Batch Incubations

Short-term colonic batch incubations were performed to simulate the proximal colon of three healthy adults as previously described by Van den Abbeele et al. [25] with minor modifications. Briefly, 13 mL of concentrated colonic background medium (25.2 g/L K_2_HPO_4_, 79.0 g/L KH_2_PO_4_, 9.7 g/L NaHCO_3_ (Chem-Lab NV, Zedelgem, Belgium), 9.7 g/L yeast extract, 9.7 g/L peptone (Oxoid, Aalst, Belgium), 4.8 g/L mucin, 2.4 g/L cysteine, 9.7 g/L Tween^®^ 80 (Sigma-Aldrich, Bornem, Belgium)) was administered to 120 mL penicillin bottles already containing 50 mL of dialyzed test product (diluted with dH_2_O to a final concentration of 4 g/L (in final volume of 70 mL)). This medium was previously demonstrated to facilitate growth of a broad spectrum of microbes belonging to various phyla (Actinobacteria, Bacteroidetes, Firmicutes, and Proteobacteria) [27]. Additionally, for each donor, a reference ‘no substrate control’ incubation was initiated simultaneously. The advantage of comparing such a ‘no substrate control’ is that any changes observed between this condition and the BP-treated condition can be attributed to BP treatment. All reactors were sealed with a rubber stopper and flushed with nitrogen to remove oxygen prior to inoculation. 

Fresh fecal samples were collected from three healthy adults and immediately stored in an airtight container with an AnaeroGen^®^ sachet (Oxoid). Fecal samples were stored at 4 °C in the anaerobic container until further processing. Fecal inocula were prepared by making a 7.5% (*w*/*v*) suspension of each of the freshly collected fecal samples with anaerobic phosphate buffer (8.8 g/L K_2_HPO_4_, 6.8 g/L KH_2_PO_4_, 0.1 g/L sodium thioglycolate, 0.015 g/L sodium dithionite). After homogenization (10 min, BagMixer 400, Interscience, Louvain-La-Neuve, Belgium) and removal of large particles via centrifugation (2 min, 500× *g*), 7 mL of inoculum was added to each penicillin bottle, yielding a total volume of 70 mL inside each reactor. Once the inoculum was added, the incubation started and lasted for a period of 48 h. Bottles were maintained at 37 °C and were continuously mixed in a temperature-controlled shaker (90 rpm). Given the high technical reproducibility of the assay as demonstrated before [28], it was opted to test three donors in single repetition rather than testing one donor in technical triplicate. This approach allowed understanding potential interpersonal differences. While samples for the analysis of fermentation products were collected at 0, 6, 24, and 48 h, samples for the analysis of specific taxonomic groups were collected at 0, 24, and 48 h.

### 2.4. Microbial Metabolic Activity

The extent of acidification during the short-term incubations is a measure for the degree of bacterial fermentation activity. The pH was measured immediately using a SenseLine F410 (ProSense, Oosterhout, The Netherlands). As the incubations were performed in a closed incubation system, one could determine the accumulation of gases in the headspace by penetrating the rubber septum with a needle connected to a pressure meter (Hand-held pressure indicator CPH6200; Wika, Echt, The Netherlands). Furthermore, short-chain fatty acids (SCFAs) and branched-chain fatty acids (bCFAs) were quantified by gas chromatography (GC) coupled to flame ionization detection (FID) as previously described by De Weirdt et al. [29]. Lastly, lactate was quantified using a commercially available enzymatic assay kit (R-Biopharm, Darmstadt, Germany) according to the manufacturer’s instructions. All these aforementioned endpoints do not determine the instantaneous microbial activity, yet they reflect the activity during the preceding incubation period.

### 2.5. Quantification of Specific Taxonomic Groups

Luminal samples were subjected to quantitative polymerase chain reaction (qPCR) to quantify specific populations of the simulated human gut microbiota. DNA isolation and qPCR analyses were performed as described by Van den Abbeele et al. [28]. Briefly, a StepOnePlus™ real-time PCR system (Applied Biosystems, Foster City, CA, USA), was used to quantify five taxonomic groups of interest, i.e., *Lactobacillus* spp. [30], *Bifidobacterium* spp. [31], *Akkermansia muciniphila* [32], *Bacteroidetes* [33], and *Firmicutes* [33]. All protocols started with 10 min incubation at 95 °C and terminated with a melting curve from 60 °C to 95 °C. Forty cycles were performed with a denaturation phase of 15 s at 95 °C, an annealing phase of 30 s at 60 °C, and an elongation step of 30 s at 72 °C in each cycle. The primers used are presented in Table 1.

### 2.6. Statistics

For exploratory data analysis, principal component analysis (PCA) was performed for both metabolic (acidification, acetate, propionate, butyrate, and bCFAs) and qPCR data via the online tool Clustvis (https://biit.cs.ut.ee/clustvis/, accessed on 7 May 2021) [34]. To statistically evaluate differences in microbial metabolite production between ‘no substrate control’ and treatment incubations at each timepoint, two-way paired Student *t*-tests were performed. Differences were considered significant when *p* < 0.05, although different levels of significance were distinguished: * *p* < 0.05, ** *p* < 0.01. Statistical analysis was performed with Microsoft Excel (version 365, Microsoft, Redmond, WA, USA). 

## 3. Results

### 3.1. Baobab Fruit Pulp Powder Stimulated Microbial Metabolic Activity from 0–24 h with Some Interindividual Differences 

**Figure 2:**
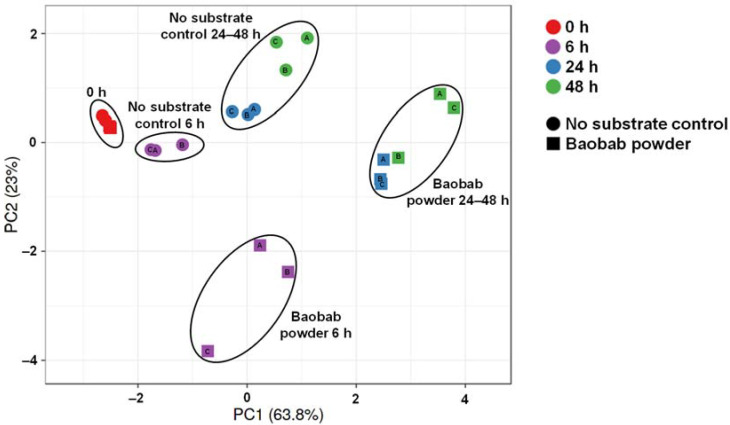
Principal component analysis (86.8%) of the metabolic activity data recorded along the 48 h incubations (pH, gas, acetate, lactate, propionate, butyrate, bCFA). Different symbol shapes indicate different conditions (‘no substrate control’ vs. baobab fruit pulp powder), while different colors reflect different timepoints. Letters inside symbols refer to the respective donor (A, B, or C).

To gain insight into the overall changes in microbial activity upon BP treatment, a principal component analysis (PCA) was performed (Figure 2). The PCA accounted for 86.6% of the observed variation of the dataset, thus providing optimal insight into the underlying changes. First, differential clustering of ‘no substrate control’ versus BP-treated incubations indicated the occurrence of treatment effects. Furthermore, a marked time-course effect was observed with main fermentation of BP occurring between 0 and 24 h. Interestingly, within clusters of time/treatment, interindividual differences among the three donors were observed.

### 3.2. Baobab Fruit Pulp Powder Stimulated Acetate, Propionate, Lactate, and Butyrate Production

**Figure 3:**
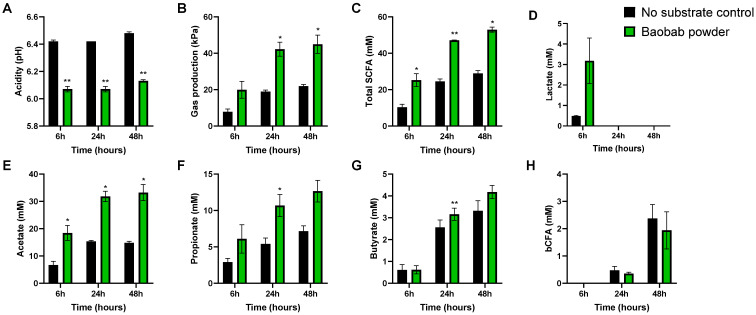
Effect of baobab fruit pulp powder fermentation on microbial fermentation products (**A**–**H**) during 48 h incubations. Average (±SEM) values across the three donors tested (*n* = 1 per donor) at 6 h, 24 h, and 48 h of incubations for the ‘no substrate control’ (black) and upon treatment with baobab fruit pulp powder (green). Statistically significant differences are indicated with asterisks (* *p* < 0.05, ** *p* < 0.01).

The average changes in microbial metabolic activity across donors allowed identifying potential consistent effects of BP (Figure 3). Gas production, acidification (pH), and total SCFA production are considered general fermentation markers and were consistently impacted by BP within 6 h after initiation of the incubation. The pH decrease and increase in total SCFA levels with BP was due to the marked stimulation of mostly acetate and propionate. Furthermore, butyrate only mildly increased, still reaching significance at the 24 h timepoint. pH decreases were further explained by the stimulatory effect of BP on lactate production. Although nonsignificant (*p* = 0.14), lactate was increased approximately sixfold upon treatment after 6 h of incubation. Lastly, BP tended to reduce the production of bCFAs at 48 h (*p* = 0.14). 

**Figure 4:**
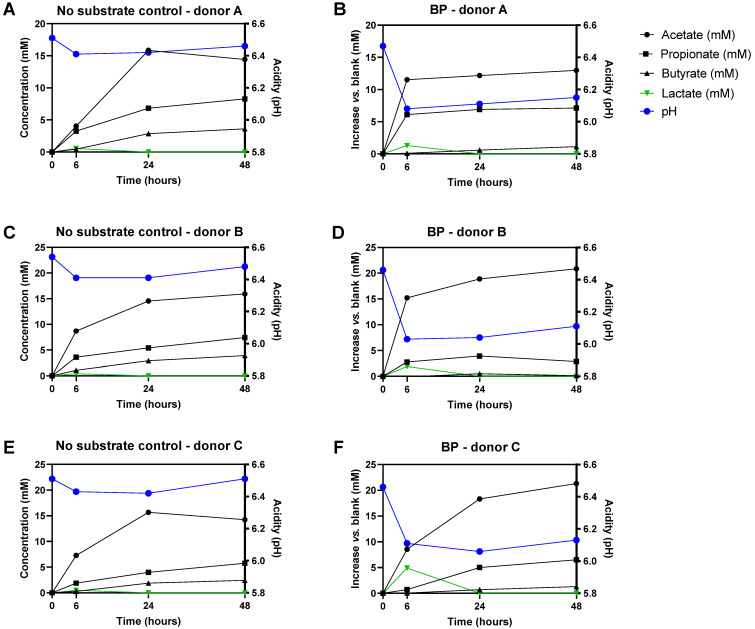
Temporal changes in microbial metabolic activity markers (acetate, propionate, butyrate, lactate, and pH) for three different healthy adult donors (donor A, donor B, donor C) during 48 h fecal batch incubations for the ‘no substrate control’ (black) and upon treatment with baobab fruit pulp powder (right) (**A**–**F**). Temporal profiles of reference conditions indicate absolute values while profiles of treatments with baobab fruit pulp powder represent changes as compared to the reference conditions.

Upon plotting the kinetics of metabolite production for each individual donor (Figure 4), interindividual differences were visualized. This pointed out that BP strongly increased acetate and propionate levels within 6 h for donors A/B, while rather increasing lactate levels for donor C at this time point. Acetate, propionate, and butyrate production was rather delayed to the 6–24 h time interval for this donor.

In terms of overall kinetics, a peculiar finding was that lactate was exclusively detected at the initial 6 h timepoint in all incubations. The transient nature of lactate is likely explained through cross-feeding mechanisms in which lactate is consumed for the production of, e.g., acetate, propionate, and/or butyrate. 

### 3.3. Baobab Fruit Pulp Powder Altered the Abundance of Specific Members of the Microbial Community in a Donor-Dependent Fashion

**Figure 5:**
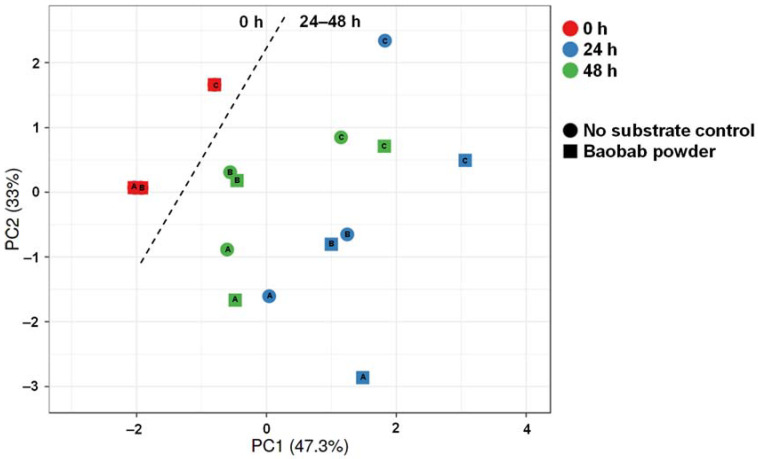
Principal component analysis (80.3%) based on the quantification of five specific taxonomic groups during the 48 h incubations. qPCRs targeted *Bifidobacteria*, *Lactobacilli*, *Bacteroidetes*, *Firmicutes*, and *Akkermansia muciniphila*. Different symbol shapes indicate different treatments (‘no substrate control’ vs. baobab fruit pulp powder), while different colors indicate different timepoints. Letters inside symbols refer to the respective donor (A, B, or C).

The PCA-based quantification of specific microbiota members (*Bifidobacteria*, *Lactobacilli*, *Bacteroidetes*, *Firmicutes*, and *Akkermansia muciniphila*) again explained a high degree of variation (80.3%, Figure 5). At the start of the experiment (0 h), donors A and B comprised similar levels of the targeted taxonomic groups, while being distinctly different from donor C. Overall, weak clustering of corresponding time/treatment samples for the three different donors indicated that interindividual variation was more profound with regard to community composition as compared to metabolic activity (Figure 2). Metabolic functional redundancy between distinct bacterial groups could explain this observation.

In terms of a treatment effect, when considered within a given donor, BP administration strongly impacted the five targeted taxonomic groups at 24 h of donors A and C, but not of donor B, as compared to their respective ‘no substrate control’ incubations. As a remark, differences versus 0 h were more pronounced at 24 h than at 48 h. 

**Figure 6:**
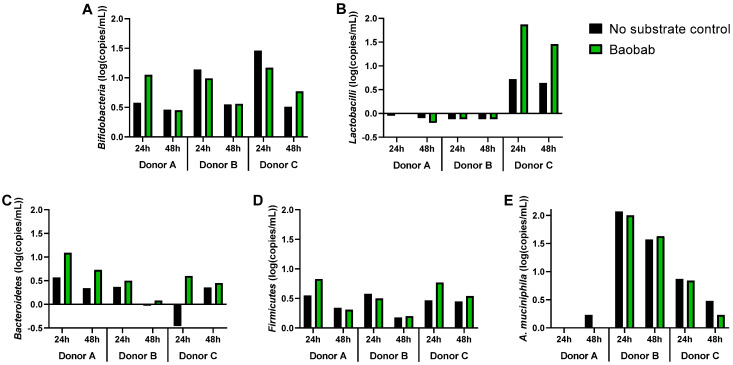
Effect of baobab fruit pulp powder on five specific taxonomic groups during 48 h incubations as determined through qPCR. Changes in absolute abundances (log (16*S* rRNA gene copies/mL)) of *Bifidobacteria* (**A**), *Lactobacilli* (**B**), *Bacteroidetes* (**C**), *Firmicutes* (**D**), and *Akkermansia muciniphila* (**E**) after 24 h and 48 h of incubation as compared to 0 h upon dosing of baobab fruit pulp powder (green) versus a ‘no substrate control’ (black) for three healthy adults (donor A, donor B, donor C) (*n* = 1).

A first observation when inspecting the underlying qPCR data (Figure 6) was that cell densities increased between 0 and 24 h and decreased between 24 and 48 h. This may indicate cell death and lysis between 24 and 48 h. After 24 h of incubation, BP stimulated *Bifidobacteria*, *Bacteroidetes*, and *Firmicutes* for donor A, while it only enriched *Bacteroidetes* for donor B. Meanwhile, for donor C, baobab fruit pulp powder strongly increased *Lactobacilli* and *Bacteroidetes* levels and moderately stimulated *Firmicutes*. Consistent with the metabolic data, these findings confirm a donor-dependent treatment response, a part of the consistent stimulation of *Bacteroidetes* members (Figure 4). 

## 4. Discussion

The present study investigated the prebiotic potential of baobab fruit pulp powder, rich in pectin-based fibers with a low degree of methyl esterification and consisting mainly of homogalacturonan (HG). This composition is unique versus other pectic polysaccharides. Thus, 48 h in vitro incubations with fecal microbiota of three human adult donors were performed to investigate the potential selective utilization of baobab fruit pulp fiber by host microorganisms, a first essential feature to qualify as a prebiotic [14]. A second essential feature is that a health benefit should follow from such selective utilization. While health effects are to be demonstrated in the final target host, health-promoting metabolites (SCFAs) were quantified during the current study to obtain first insights. Overall, this exploratory study demonstrated that baobab fruit pulp powder displays promising prebiotic potential. Depending on the donor, distinct microbial communities were present, confirming the importance of assessing interindividual variation.

First, baobab fruit pulp powder consistently stimulated specific health-related metabolites, i.e., mostly acetate and propionate. Other consistent changes included the increase in lactate levels at the start of the incubation (6 h), followed by increased butyrate levels (24 h). Furthermore, there was a tendency to lower levels of bCFA. The stimulation of acetate and propionate is in line with other studies that investigated pectin-rich fruit fibers [35,36]. This specific modulation of metabolite production suggests a specific utilization of baobab fruit pulp powder by host microorganisms able to produce such metabolites, thus confirming its prebiotic potential. Additionally, given the well-documented health benefits of acetate, propionate, and butyrate as reviewed by Rivière et al. [37], this also suggests that baobab fruit pulp powder could confer health benefits upon its consumption. bCFAs are on the other hand indicative for proteolytic fermentation [38], which is associated with formation of metabolites such as phenol and indole that exert detrimental health effects [39,40]. The tendency to lower bCFA levels upon baobab treatment, thus, further supports potential beneficial effects of baobab fruit pulp powder supplementation.

Investigation of changes in five specific taxonomic groups further confirmed the selective utilization of baobab fruit pulp powder by specific host microorganisms. Key contributors to baobab fruit pulp powder fermentation likely included *Bacteroidetes* members that increased for each of the three donors tested. This is in line with the finding that *Bacteroides* spp. possess a versatile enzymatic potential, allowing them to depolymerize the backbone of HG [19], a key constituent of baobab fruit pulp powder. As *Bacteroides* spp. are among the most abundant producers of propionate [10,41], their involvement in baobab fruit pulp powder fermentation is further supported as propionate indeed increased upon baobab fruit pulp powder supplementation. Depolymerization of HG by *Bacteroides* spp. likely facilitated fermentation of degradation fragments of HG by other microbial groups. According to the current study, such contributing microbes seem to differ among donors. Donor A was, for example, the only donor for which *Bifidobacteria* increased. In contrast, for donor C, a marked increase in *Lactobacilli* was noted, which coincided with profound increases in lactate, i.e., the sole and main end product of carbohydrate metabolism by *Lactobacilli* [42]. Stimulation of *Lactobacilli* by pectin has indeed been reported before [43]. Moreover, when mixing baobab fruit powder with fermented soybeans (Tempeh—traditional Japanese fermented food), an enhancement of lactic acid bacteria was observed [44]. While baobab fruit pulp powder increased lactate levels for all donors at 6 h, lactate was fully consumed at subsequent timepoints, indicating that baobab fruit pulp powder stimulated cross-feeding interactions with lactate-consuming microorganisms, potentially including propionate [45] and/or butyrate-producing [46] *Firmicutes* members, a phylum that indeed increased for donors A and C. Overall, these findings, even if only based on qPCR analysis (that has low taxonomic resolution as opposed to next-generation sequencing), suggest the involvement of specific host microorganisms in the fermentation of baobab fruit pulp powder, highlighting its prebiotic potential. Future research should, however, account for the marked interindividual differences among human subjects, which were apparent even after testing as few as three donors in the current study. The existence of marked interindividual differences is in line with observations during human in vivo studies [9,10].

**Figure 7:**
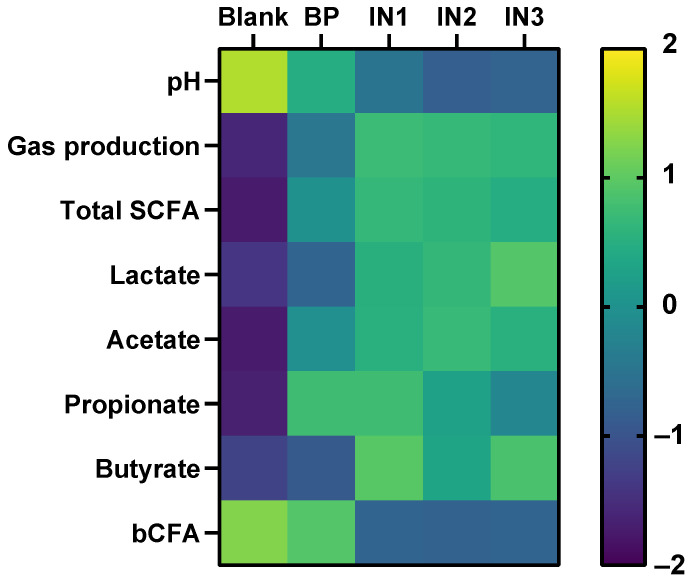
Effect of baobab fruit pulp powder (BP) fermentation on bacterial metabolic parameters during 48 h fecal batch incubations of three healthy adults compared to a ‘no substrate control’ control and three different types of inulin (IN1, IN2, IN3) as tested by Van den Abbeele et al. [25]. BP data were obtained as disclosed in this study. BP was administered at ~2.6 g fiber/L, while inulin was tested at 5 g/L. Average differences between treated and ‘no substrate control’ incubations for three donors were calculated for each parameter. Subsequently, all values were standardized within each parameter to enable comparisons across parameters on a single scale.

Now that the prebiotic potential of BP was confirmed in this first study, a next research question is how this potential relates to known prebiotic substrates. To put the findings of this study in perspective, the results were compared to those of a recent study where three different types of inulin, the ‘gold standard’ prebiotic [47], were tested using the exact same in vitro approach [25] (Figure 7). However, a key difference between both studies was that, while inulin sources were tested at 5 g/L, baobab fruit pulp powder was administered at dose of 4 g/L of which up to 33% (simple sugars) was removed upon preceding dialysis (Table S1), resulting in a colonic test dose of only ~2.6 g fiber/L. Despite being dosed at around half the dose of inulin, baobab fruit pulp powder exerted similar (IN1) or even more profound effects on propionate production (IN2/IN3). Furthermore, effects on acetate and total SCFA were more attenuated but still on the same order of magnitude. In contrast, gas production, lactate, and mostly the increase in butyrate and decrease in bCFA levels were more specific to inulin. In comparison to such ‘gold standard’ prebiotics, baobab fruit pulp powder, thus, seems an interesting potential prebiotic with a likely complementary mode of action. The marked propionate production with more attenuated gas production could be of particular interest for specific applications. It will be important to confirm these findings in studies in which BP is directly compared with known prebiotic substrates such as inulin.

## 5. Conclusions

In conclusion, this exploratory in vitro study allowed attributing an interesting prebiotic potential to baobab fruit pulp powder with changes in both fermentation products and specific taxonomic groups, suggesting selective fermentation by host microorganisms. Although subject to interindividual variation at the microbial composition level, baobab fruit pulp powder constituently stimulated the production of health-related acetate, propionate, and to lesser extent butyrate. These effects were seemingly distinctly different from those exerted by the ‘gold standard’ prebiotic inulin that rather increases butyrate production. To our knowledge, this is the first evidence demonstrating the potential of baobab fruit pulp powder to modulate the human gut microbiota. Overall, our findings strongly support further research toward the potential of baobab fruit pulp powder as a prebiotic substrate. Such studies should account for the investigation of effects across multiple donors given the observed interindividual variation during the current study. Performing in vitro studies with a fecal inoculum of a single human donor could result in conclusions that are not representative of a broader number of donors. For example, if only donor C was tested, the conclusion would have been that baobab fruit pulp powder strongly stimulates *Lactobacilli*. While valid for donor C, this was not the case for donors A/B. Overall, this study supports further research toward the prebiotic potential of baobab fruit pulp powder and other pectin-based products, as well as their potential health-promoting effects.

## Figures and Tables

**Table 1:** Primers used for qPCR quantification of 5 specific taxonomic groups.

Taxonomic Group	Primer Sequences 5′–3′ and 3′–5′	Reference
*Lactobacillus* spp.	AGCAGTAGGGAATCTTCCA CGCCACTGGTGTTCYTCCATATA	[30]
*Bifidobacterium* spp.	TCGCGTCYGGTGTGAAAG CCACATCCAGCYTCCAC	[31]
*Akkermansia muciniphila*	CAGCACGTGAAGGTGGGGAC CCTTGCGGTTGGCTTCAGAT	[32]
*Bacteroidetes*	GGAACATGTGGTTTAATTCGATGAT AGCTGACGACAACCATGCAG	[33]
*Firmicutes*	GGAGYATGTGGTTTAATTCGAAGCA AGCTGACGACAACCATGCAC	[33]

## Supplementary Materials

The following are available online at https://www.mdpi.com/article/10.3390/microorganisms9091981/s1, Table S1. Nutritional composition of tested baobab fruit pulp powder.
