# Supplementary material for: A Pectin-Rich, Baobab Fruit Pulp Powder Exerts Prebiotic Potential on the Human Gut Microbiome In Vitro
Source: Microorganisms. 2021 Sep 17;9(9):1981. doi: 10.3390/microorganisms9091981 (PMC8467054; doi:10.3390/microorganisms9091981)
Supplement: Supplementary file 1 [file microorganisms-09-01981-s001.zip › microorganisms-1339024-supplementary.pdf]

microorganisms

Table S1. Nutritional composition of tested baobab fruit pulp powder. TDF = total dietary fiber, WSS-PS = water soluble solid-polysaccharide, WUS-PS = water unextractable solids.

Parameter	Baobab Powder
Dry mass (%)	93
Starch (% of dry mass)	2.7
Starch (% of total mass)	2.5
Protein (% of dry mass)	2.8
Protein (% of total mass)	2.6
Glucose, fructose, sucrose (% of dry mass)	35
Glucose, fructose, sucrose (% of total mass)	33
TDF (% of dry mass)	66
WSS-PS (% of dry mass)	50.5
WSS-PS (% of total mass)	47
WUS-PS (% of dry mass)	13
Pectin (% of dry mass)	42.5
